# Ultrasound-triggered oxygen-loaded nanodroplets enhance and monitor cerebral damage from sonodynamic therapy

**DOI:** 10.7150/ntno.71946

**Published:** 2022-06-27

**Authors:** Harriet Lea-Banks, Sheng-Kai Wu, Hannah Lee, Kullervo Hynynen

**Affiliations:** 1 Physical Sciences Platform, Sunnybrook Research Institute, Toronto, Canada.; 2 Department of Medical Biophysics, University of Toronto, Toronto, Canada.; 3 Institute of Biomedical Engineering, University of Toronto, Toronto, Canada.

**Keywords:** ultrasound, cavitation, phase-change emulsion, microbubble

## Abstract

In sonodynamic therapy, cellular toxicity from sonosensitizer drugs, such as 5-aminolevulinic acid hydrochloride (5-ALA), may be triggered with focused ultrasound through the production of reactive oxygen species (ROS). Here we show that by increasing local oxygen during treatment, using oxygen-loaded perfluorocarbon nanodroplets (250 +/- 8 nm), we can increase the damage induced by 5-ALA, and monitor the severity by recording acoustic emissions in the brain. To achieve this, we sonicated the right striatum of 16 healthy rats after an intravenous dose of 5-ALA (200 mg/kg), followed by saline, nanodroplets, or oxygen-loaded nanodroplets. We assessed haemorrhage, edema and cell apoptosis immediately following, 24 hr, and 48 hr after focused ultrasound treatment. The localized volume of damaged tissue was significantly enhanced by the presence of oxygen-loaded nanodroplets, compared to ultrasound with unloaded nanodroplets (3-fold increase), and ultrasound alone (40-fold increase). Sonicating 1 hr following 5-ALA injection was found to be more potent than 2 hr following 5-ALA injection (2-fold increase), and the severity of tissue damage corresponded to the acoustic emissions from droplet vaporization. Enhancing the local damage from 5-ALA with monitored cavitation activity and additional oxygen could have significant implications in the treatment of atherosclerosis and non-invasive ablative surgeries.

## Introduction

Over the last two decades, clinical approval of 5-aminolevulinic acid hydrochloride (5-ALA) as an intra-operative diagnostic tool during glioma resection has been granted in over 40 countries, firstly in Europe (2007) and now across North America (2017) 1. The optical properties of 5-ALA allow residual tumour tissue to be visualized with fluorescence imaging and surgically removed. Preclinical work has also investigated 5-ALA as a therapeutic tool in photodynamic therapy (PDT), where tumour-specific toxicity can be induced, triggered with laser light, in the treatment of skin 2 and prostate 3 cancers.

In 1993, the term 'sonodynamic' was first published to describe how porphyrin can be activated by ultrasound to elicit cytotoxic effects 4. More recently, sonodynamic therapy (SDT) has been achieved with 5-ALA, by triggering toxicity with MRI-guided focused ultrasound (FUS) in the brain; it has shown promise in treating glioma in preclinical models 5, and is currently under clinical investigation (NCT04559685). Focused ultrasound can reach targets deep within tissue, beyond the penetration depth of light, and therefore offers an attractive alternative for brain therapies. Although the underlying mechanisms of sonodynamic therapy are not fully understood, they are proposed to be driven by sonoluminescence and the generation of reactive oxygen species (ROS) 6, both of which occur during violent bubble collapse, known as inertial cavitation.

However, SDT can also be achieved at very low ultrasound pressures (0.1-0.2 MPa, 1 MHz), shown in combination with the sonosensitizers indocyanine green 7 and DCPH-P-Na(I) 8, without requiring inertial cavitation for the generation of ROS and cytotoxicity. Therefore, it is likely that direct ultrasound-mediated generation of ROS through inertial cavitation (mechanism 1), and ROS generation through sonoluminescence from non-inertial or inertial cavitation (mechanism 2), both contribute to SDT 9, and that the production of ROS is increased when ultrasound is used to activate a sonosensitizer 10.

5-ALA is a precursor of Protoporphyrin IX (PpIX), a potent sonosensitizer that develops as the drug is metabolized within the cell before being converted to heme. Therefore the time window of 5-ALA toxicity is determined by the accumulation and conversion rate of 5-ALA to PpIX, reported to be between 6-8 hr after injection in an osteosarcoma mouse model 11 and recently in human glioma 12. Build-up of PpIX occurs in cancer cells due to incomplete heme synthesis. Although this build up is not seen to the same extent in normal cells, there is a window when 5-ALA is converted to PpIX before becoming heme, which could open the door to non-invasive surgical ablation in non-cancerous tissue. This effect has been exploited in the treatment of atherosclerosis in a rodent model 13, where the toxicity was found to be greatest 1 hr post-injection.

Since 5-ALA is less potent to non-cancerous tissue, we hypothesize that introducing additional oxygen during sonication may increase the production of ROS, by providing a greater amount of available molecular oxygen at the ultrasound focus during sonication. Furthermore, to exploit both direct ultrasound-mediated ROS generation (mechanism 1), and sonoluminescence-based ROS generation (mechanism 2), we use a cavitation agent as the oxygen carrier to further increase the likelihood of ROS production through non-inertial and inertial cavitation.

Perfluorocarbon (PFC) emulsions have a high capacity for dissolving oxygen and have been used as synthetic blood substitutes to transport oxygen around the body 14. PFC emulsions also respond to ultrasound, and undergo a phase-change from liquid to gas in response to a sufficient ultrasound peak negative pressure or change in temperature 15. In fact, PFC emulsions have such a high oxygen affinity they have been shown to scavenge gas during ultrasound-triggered vaporization 16. In contrast, pre-loading PFC agents with oxygen enables ultrasound-triggered release, and has been shown in nanoparticle formulations 17-19; high-boiling-point PFC nanodroplet formulations for photoacoustic therapy 20 and diagnostics 21; PFC microcapsules for chemotherapy 22; and microbubble formulations for sonodynamic therapy 23,24.

Low-boiling point PFC nanodroplets offer an attractive alternative, combining the extended circulation time of a nanoscale agent 25 with the unique acoustic behaviour of a microbubble 26, achieved at relatively low ultrasound pressures (known as the vaporization threshold). Low-boiling point nanodroplets (formed from PFCs with a boiling point below 37 °C) comprise a super-heated core which is pressurized due to the Laplace pressure, and thus locked in a liquid state even at physiological temperatures. When exposed to ultrasound, nanodroplets vaporize from liquid nanoparticles into gas microbubbles, and can deliver their cargo with high spatial precision in the brain whilst generating acoustic emissions 27.

Once a microbubble is nucleated from a droplet through vaporization, it may oscillate in a stable manner, recondense to a liquid under the positive pressure phase, or collapse under the inertia of the displaced surrounding fluid. These behaviours have been captured recently with high-speed imaging by Wu *et al.* 28. Echogenic gas bubbles scatter the fundamental frequency; oscillating bubbles can generate harmonic emissions (multiple integers of the fundamental frequency) and sub-harmonic emissions (half-multiple integers of the fundamental); collapsing bubbles can generate broadband emissions. For droplets, the onset of vaporization and inertial cavitation occur at distinct pressures, dependent on droplet size 29,30, droplet composition and concentration 31, ultrasound frequency and pulse length 32,33, ambient temperature 34 and ambient pressure 35. The pressures required for vaporization and inertial cavitation become more similar for smaller droplets and at lower sonication frequencies 32,36. The influence of these parameters on droplet vaporization have been reviewed in detail previously 15.

Here we report how low-boiling point PFC nanodroplets can be formulated by repurposing a commercially available microbubble contrast agent, can be loaded with oxygen, and can enhance SDT in the brain. We explore the role of additional molecular oxygen, alongside acoustic cavitation activity, in enhancing tissue damage in the presence of a sonosensitizer. We discuss the potential to use nanodroplets to monitor and control therapy by detecting acoustic emissions during vaporization, which could be used to inform the ultrasound pressure required in future studies. SDT has so far been underutilized in the ablation of healthy tissue but may offer a new approach for non-invasive ablative brain procedures, such as the treatment of epilepsy.

## Methodology

### Materials

5-aminolevulinic acid (5-ALA) was purchased from Millipore Sigma, USA, and stored away from light at room temperature before use. Decafluorobutane (DFB) gas (C_4_F_10_) was purchased from Synquest Labs, USA. Definity, a commercially available FDA-approved ultrasound contrast agent, was purchased from Lantheus Medical Imaging, USA. In its native form, Definity is a lipid-based microbubble solution filled with octafluoropropane (OFP) (C_3_F_8_). The lipid shell is composed of (R)-4-hydroxy-N,N,N-trimethyl-10-oxo-7-[(1oxohexadecyl)oxy]-3,4,9-trioxa-4-phosphapentacosan-1-aminium, 4-oxide, (DPPC); (R)-hexadecanoic acid, 1-[(phosphonoxy)methyl]-1,2-ethanediyl ester (DPPA); and (R)-∝-[6-hydroxy-6-oxido-9-[(1-oxohexadecyl)oxy]-5,7,11-trioxa-2aza-6-phosphahexacos-1-yl]-ω-methoxypoly(ox-1,2-ethanediyl) (MPEG5000 DPPE), at a molar ratio of 82:10:8, at a total lipid concentration of 0.75 mg/mL. Milli-Q ultrapure water (Millipore Sigma, US) and medical grade pure oxygen were used throughout the fabrication. Modified Winkler titrations were performed using reagents from HANNA Instruments, USA.

### Oxygen-loaded perfluorocarbon nanodroplets

Low-boiling point nanodroplets were formed by the condensation method (37). 1.5 mL of the Definity lipid solution was degassed in a sealed 3 mL-capacity Wheaton vial for 30 min with frequent agitation to remove the native OFP (boiling point -37 °C), and the headspace of the vial was filled with DFB (boiling point -2 °C). DFB-Definity precursor microbubbles were formed by 45 s of agitation with the VialMix (Lantheus Medical Imaging, USA) shaker, and then condensed in a bath of isopropanol cooled to -10 °C with dry ice. The pressure inside the vial was increased with the addition of 1 mL of atmospheric air to promote microbubble condensation, creating a clear droplet solution. The remaining precursor microbubbles were removed by centrifugal washing (300 G, 8 min) at 4 °C, and sub-micron droplets were size-isolated by slowly passing the solution through a cold syringe filter (0.8 µm pore size, Minisart syringe filter, Sartorius, Germany) on ice. Definity-derived nanodroplets were then loaded with oxygen by slowly bubbling a fixed volume of pure oxygen through the suspension on ice prior to use. Nanodroplet size was assessed with dynamic light scattering (ZetaSizer, Malvern Instruments, UK), and size stability was assessed by measuring the mean hydrodynamic diameter and polydispersity index over 6 hr whilst stored on ice.

A modified Winkler method 38 was used to quantify the amount of dissolved oxygen in suspensions of oxygen-loaded nanodroplets, compared to unloaded nanodroplets and ultra-pure Milli-Q water, after bubbling fixed volumes of oxygen (0-30 mL at a rate of 10 mL/min) at atmospheric pressure through the samples at 4 °C. To perform the Winkler titration, manganese sulphate solution was added to samples of Milli-Q or nanodroplets in Milli-Q in air-tight tubes. The addition of a potassium iodide-azide reagent created a precipitate of manganese oxide, and the sample was violently vortexed for 5 min at maximum speed to intentionally vaporize the droplets, disrupt the phospholipid shell, and release the dissolved oxygen. Sulphuric acid was used to reduce the solution, and a starch indicator was used to titrate the resulting iodine. Samples and controls were performed simultaneously and in triplicate, ensuring the sample tubes were entirely full to displace all air and to avoid introducing additional oxygen.

Spectrophotometric titrations of dissolved oxygen in water and solutions of nanodroplets were quantified with UV/Vis absorbance (Synergy H1, Biotek, USA), isolating the magnitude of the absorption peak at 350 nm. The control case (ND, 0mL of oxygen) was used to control for any additional scattering or absorbance from fragments of the lipid shell or remaining droplets. By plotting the absorbance at 350 nm against the volume of titrant, the endpoint of the titration was determined (i.e. the volume of titrant needed to complete the titration, approximately equivalent to the concentration of the titrate). 1 mL of titrant was equivalent to 10 mg/L of dissolved oxygen.

### Animal preparation, 5-ALA injection time point and dosing

16 Sprague Dawley rats (328 +/- 18 g) (Taconic Biosciences, USA) were used to assess the efficacy of oxygen-loaded nanodroplets for sonodynamic therapy, distributed between four treatment groups (Table 1). Animals were housed in the Sunnybrook Research Institute animal facility (Toronto, Canada) on a reverse light cycle and had access to food and water *ad libitum*. All animal procedures were approved by the Animal Care Committee at Sunnybrook Research Institute and are in accordance with the Canadian Council on Animal Care and ARRIVE guidelines.

Before treatment, anaesthetic induction was achieved with 5% isoflurane with medical air and reduced to 2% isoflurane for preparation and treatment. Scalp hair was removed with clippers and depilatory cream, and a 22-gauge catheter was inserted into the tail vein. All injections were administered intravenously. The sonosensitizer 5-ALA (Millipore Sigma, US) was dissolved in phosphate-buffered saline (PBS, pH 7.4) at a concentration of 100 mg/mL and then stored at 4 °C in the dark before use. One or two hours prior to sonication, a dose of 200 mg/kg 5-ALA was intravenously administrated via a slow bolus. Injection time point and dose were based on previous studies showing 5-ALA accumulation peaks at 1 hr-post injection in non-cancerous tissue 13.

**Figure 1:**
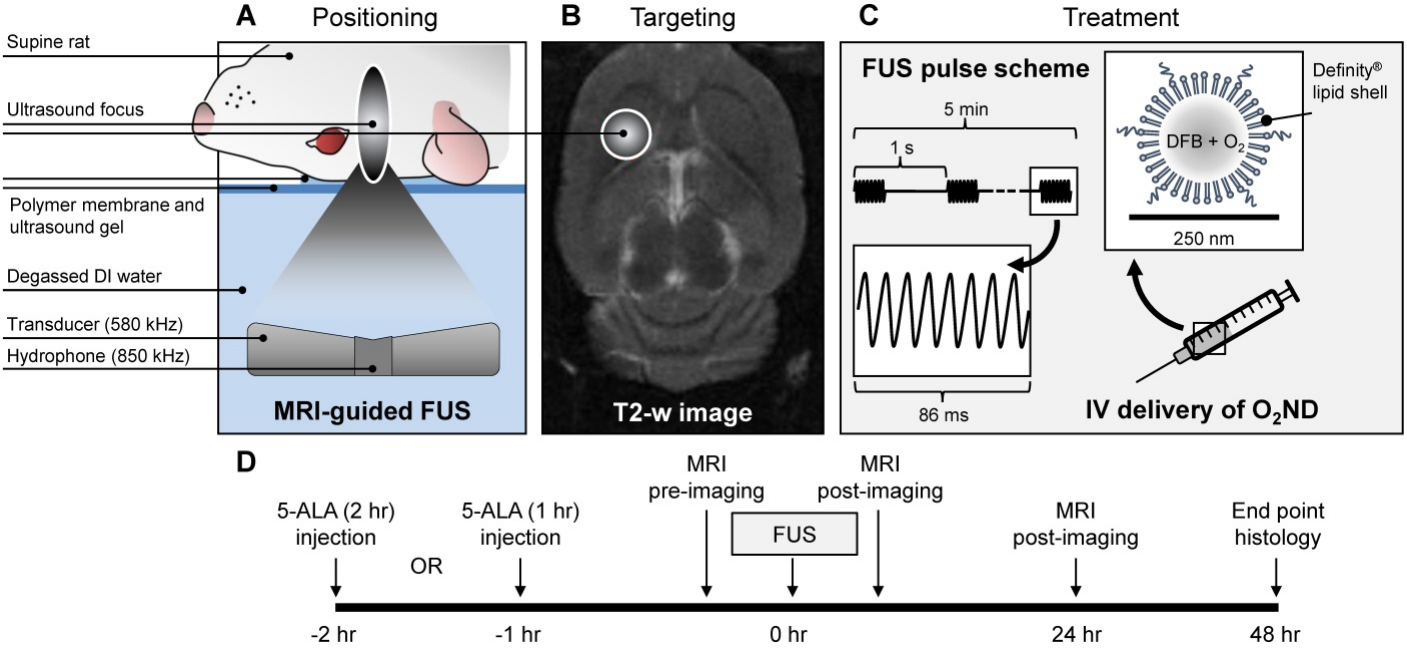
** Summary of experimental procedure. (A)** Positioning of anaesthetized rat supine on MRI-guided focused ultrasound (FUS) system, **(B)** right striatum targeted from T2-weighted MR image, **(C)** pulsed ultrasound delivered for 5 min treatment duration, with oxygen-loaded nanodroplets (O_2_ND) injected intravenously (IV), **(D)** summarized in the experimental timeline.

Rats were positioned supine on the platform of the ultrasound system, with the scalp coupled to the tank of degassed water through a thin layer of ultrasound gel and polymer membrane (Figure 1). Body temperature was maintained with warm saline bags.

### MRI-guided focused ultrasound and post-treatment imaging

MRI-guided focused ultrasound was delivered using a preclinical ultrasound system and targeted with a 7-Tesla MRI scanner (BioSpec 70/30 USR, Bruker, USA). T2-weighted coronal images were used to target the specific anatomical location. FUS was delivered at 580 kHz with a spherically focused transducer (580 kHz center frequency, 60 mm radius of curvature, 75 mm external diameter with a circular cut-out of 20 mm diameter in the center of the transducer), calibrated using a planar fiber optic hydrophone with an active tip diameter of 10 μm (Precision Acoustics Ltd., Dorset, UK). A fixed transmitted pressure of 2.0 MPa was used for 5 min in 86 ms bursts, with a pulse repetition frequency of 1 Hz, to a single target in the right striatal area, covering a focal volume of 3 × 3 × 20 mm. 0.5 mL of unloaded nanodroplets, oxygen-loaded nanodroplets or saline was slowly injected following 10 s of acoustic baseline measurements, followed by 0.2 mL saline flush. The maximum pulse length and pulse repetition frequency were constrained by the function generator (Agilent, 33220A), which could achieve a maximum of 50,000 cycles per burst (i.e. 1 s / 580,000 Hz * 50,000 cyc = 86 ms).

MR imaging was carried out immediately following treatment and 24 hours after treatment. T2-weighted imaging (4000 ms TR, 70 ms TE, 256 × 256 matrix, 1.5 mm slice thickness) and T2*-weighted imaging (800 ms TR, 3 ms TE, 256 × 256 matrix, 1.5 mm slice thickness) were used to identify edema and haemorrhage respectively. During follow-up imaging (24 hr post-treatment) the animal was positioned prone and a rat brain surface coil (T11425V3, Bruker, USA) was used to enhance the images.

MIPAV (Bethesda, MA, USA) software was used for MRI image analysis. For T2*-weighted images, hypointense regions in the striatum were manually contoured, and the resulting area was converted from pixels to mm^2^ to quantify the haemorrhagic region. Areas from each of six total slices were summed to produce the final hypointense area. For T2-weighted images, the right (treated) and left (untreated) striatum were manually contoured; the mean and standard deviation of pixel intensity were calculated for each side, and the number of pixels on the treated side where the intensity exceeded the mean intensity of the untreated side by more than 3-times the standard deviation was quantified. The resulting hyperintense area was converted from pixels to mm^2^ to quantify the region of edema. Areas from each of six total slices were summed to produce the final hyperintense area.

### Acoustic monitoring

Ultraharmonic emissions (3/2-times the fundamental transmit frequency) were captured with a narrowband lead zirconate titanate (PZT) hydrophone, tuned to 850 kHz (-6 dB bandwidth of 60 kHz, Figure 5B), housed within a central circular cut-out of the 580 kHz transmit transducer, and coaxially aligned to the focus. Data was captured at a sampling rate of 20 MHz, and spectral analysis was performed in MATLAB to assess the magnitude of ultraharmonic emissions throughout the 5 min sonication for each treatment group. Broadband emissions were quantified by calculating the area under the frequency spectra. Harmonic and ultraharmonic peaks were subtracted out using the 50% sensitivity bandwidth of the receiver to avoid signal processing sidelobes. Ultraharmonic and broadband emissions are normalized to baseline measurements acquired prior to nanodroplet injection (as has been used previously for microbubble exposures 39).

### Histology

Rats were perfused with 10% formaldehyde solution, and brains were excised 48 hr after treatment to assess haemorrhage and tissue damage. Fixed brain tissue was embedded in paraffin wax and sectioned at 5-µm thickness. Hematoxylin and eosin (H&E) and terminal deoxynucleotidyl transferase dUTP nick end labeling (TUNEL, Promega, Madison, WI, US) staining were performed to assess haemorrhage and cell apoptosis, respectively.

Brightfield microscopy images (Zeiss Axio Observer Z1; Carl Zeiss, Germany) of H&E and TUNEL stained brain sections were taken at 5x and 10x magnification. ImageJ (Bethesda, MA, USA) software was used for image analysis with standard contour and counting functions. For H&E-stained slides, the perimeter of haemorrhagic regions was manually contoured and the area (mm^2^) was calculated with ImageJ. TUNEL positive apoptotic cells were identified manually from TUNEL-stained slides.

### Statistical Analysis

Single-factor analysis of variance (ANOVAs) was performed for the area data sets for each of T2* (0 hr), T2 (0 hr), T2* (24 hr), T2 (24 hr), and H&E (48 hr) images. Significant differences in the extent of tissue damage were identified using a post-hoc T-test with a p-value of less than 0.05. Correlation between damage area and acoustic activity was assessed with exponential regression analysis, quantified with R^2^ coefficient.

## Results

### Characterization of oxygen-loaded nanodroplets

**Figure 2:**
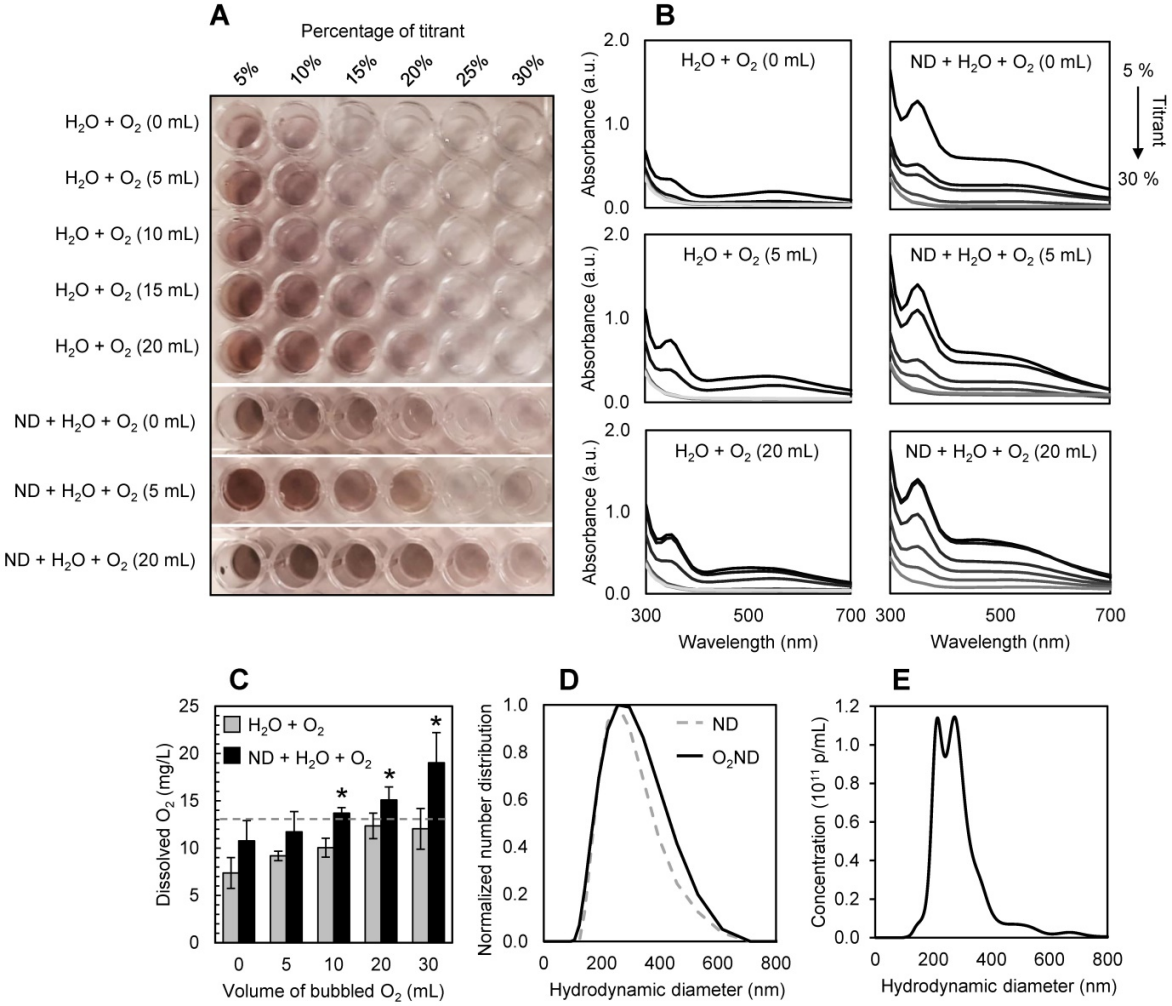
** Characterization of oxygen-loaded decafluorobutane (DFB) nanodroplets (O_2_ND). (A)** Spectrophotometric titrations of dissolved oxygen in water and solutions of nanodroplets using a modified Winkler method, and **(B)** corresponding absorbance measurements. **(C)** Super-saturation of dissolved oxygen in water (grey bars) shown with the presence of perfluorocarbon nanodroplets (black bars). Dashed line indicates the dissolved oxygen saturation point of 4°C water predicted by Henry's Law. **(D)** Dynamic light scattering used to assess nanodroplet size distribution, showing mean hydrodynamic diameter before (dashed line) and after (solid line) oxygen-loading. **(E)** Nanoparticle tracking analysis used to assess oxygen-loaded nanodroplet size and concentration distribution. Errors bars show 1 standard deviation from 3 independent samples (*p<0.05).

Nanodroplets were fabricated through condensation, with a Definity-lipid shell and DFB liquid-state core, and had a mean hydrodynamic diameter of 213 +/- 2 nm before oxygenating. After loading with oxygen, nanodroplets had a mean hydrodynamic diameter of 250 +/- 8 nm (Figure 2D), potentially driven by Ostwald ripening. No spontaneous nanodroplet vaporization (i.e. production of microbubbles) was detected following oxygen-loading, verified with microscopy and dynamic light scattering. No significant change in hydrodynamic diameter or polydispersity index was recorded after 2 hr on ice, showing comparable stability to unloaded DFB nanodroplets 33.

After washing and filtering, the final lipid concentration in the nanodroplet solution was found to be 0.29 +/- 0.07 mg/mL from three independent batches, quantified by UV-Vis absorbance at 200 nm against a standard curve of Definity lipid solution (NanoDrop 2000/2000c spectrophotometer, ThermoFisher). Filtered, washed and oxygenated DFB nanodroplets had a particle concentration of 2.1 +/- 0.2 × 10^11^ p/mL, quantified by nanoparticle tracking analysis (NTA) (NanoSight, Malvern Panalytical), equivalent to 0.2% PFC volume (Figure 2E).

The presence of perfluorocarbon nanodroplets allowed the solution to be super-saturated with oxygen, increasing the dissolved oxygen content from 13 +/- 3 mg/L (equivalent to the dissolved oxygen saturation point of water at 4 °C, as predicted by Henry's Law and verified experimentally) to 19 +/- 3 mg/L, after 30 mL of oxygen was bubbled at a rate of 10mL/min. Figure 2A shows a representative 96-well plate used for spectrophotometric titrations of dissolved oxygen, quantified with absorbance measurements (Figure 2B, 2C).

### MRI assessment of tissue damage

**Figure 3:**
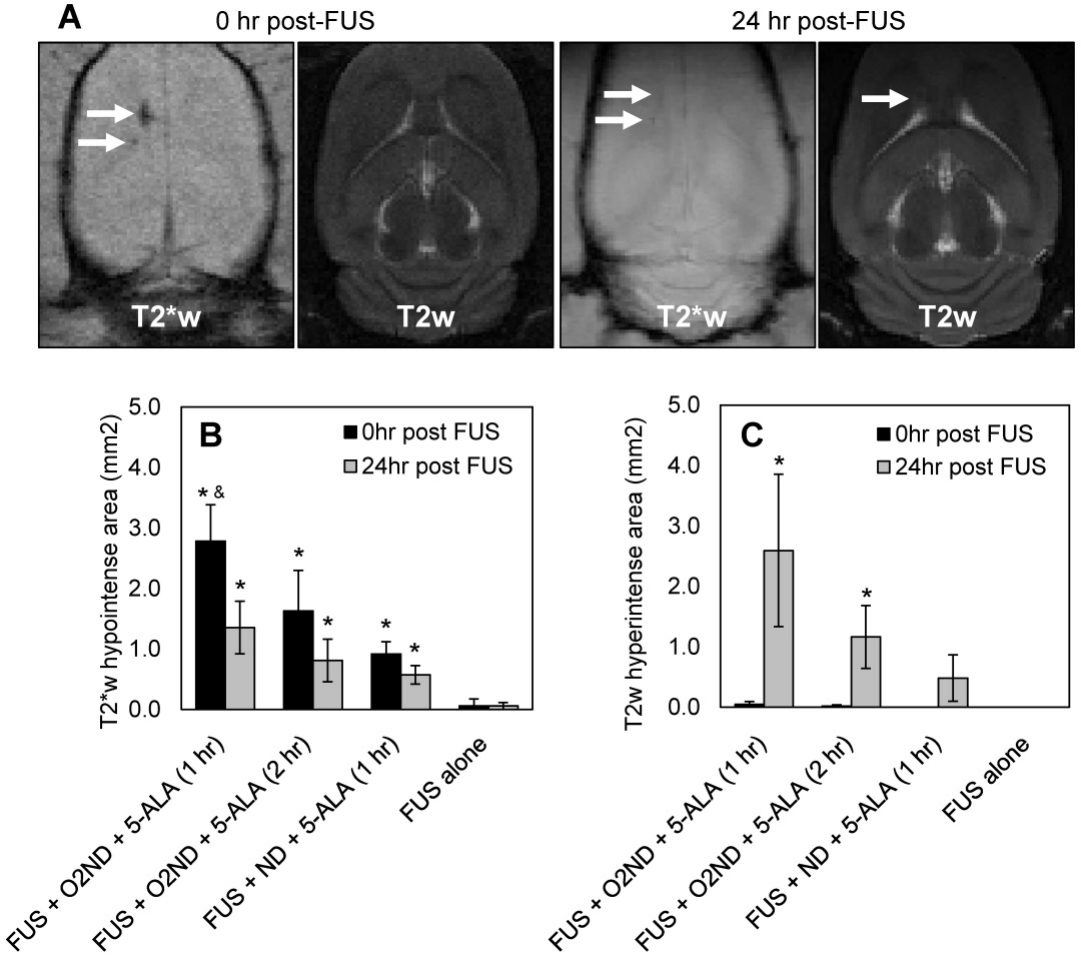
** MRI assessment of tissue damage following FUS treatment. (A)** Example MRI images from a rat treated with FUS + O_2_ND + 5-ALA (1 hr). Damage from all rats is quantified by **(B)** hypointense and **(C)** hyperintense regions on T2*- and T2-weighted images respectively. 24 hr post-FUS MRI images were acquired with a head coil for higher resolution. Each column represents data from 4 rats (1 rat was excluded from FUS + ND + 5-ALA (1 hr)), and error bars represent SEM. Significance compared to FUS alone is indicated by * (p<0.05), and significance compared to FUS + ND + 5-ALA (1 hr) is indicated by & (p<0.05).

Regions of haemorrhage and edema were measured immediately following sonication and 24 hr after sonication with MRI, using T2- and T2*-weighted scans. Figure 3A shows representative MRI images from a rat treated with FUS + O_2_ND + 5-ALA (1 hr), using a head coil to enhance the imaging 24 hr post-FUS. MRI images are quantified by hypointense areas on T2*-weighted scans (indicative of haemorrhage) (Figure 3B), and hyperintense areas on T2-weighted scans (indicative of edema) (Figure 3C). One rat was excluded from the study, treatment group FUS + ND + 5-ALA (1 hr), due to incorrect targeting.

FUS alone caused no detectable haemorrhage or edema, verified with MRI. Oxygen-loaded nanodroplets, exposed to FUS, caused greater haemorrhage and edema compared to FUS alone 24 hr following sonication in the presence of 5-ALA (Figure 3). Exposing healthy cerebral tissue to FUS and oxygen-loaded nanodroplets 1 hr after 5-ALA injection showed greater damage than 2 hr after injection. In the case of FUS + O_2_ND + 5-ALA (1 hr), a significantly larger haemorrhagic region was produced compared to FUS + ND + 5-ALA (1 hr), illustrating the role of local oxygen in facilitating 5-ALA toxicity. As expected, some of the haemorrhagic regions cleared over 24 hr (as seen on T2*-weighted images, Figure 3A, 3B), and edema increased after 24 hr (as seen on T2-weighted images, Figure 3A, 3C).

### Histological assessment of tissue damage

**Figure 4:**
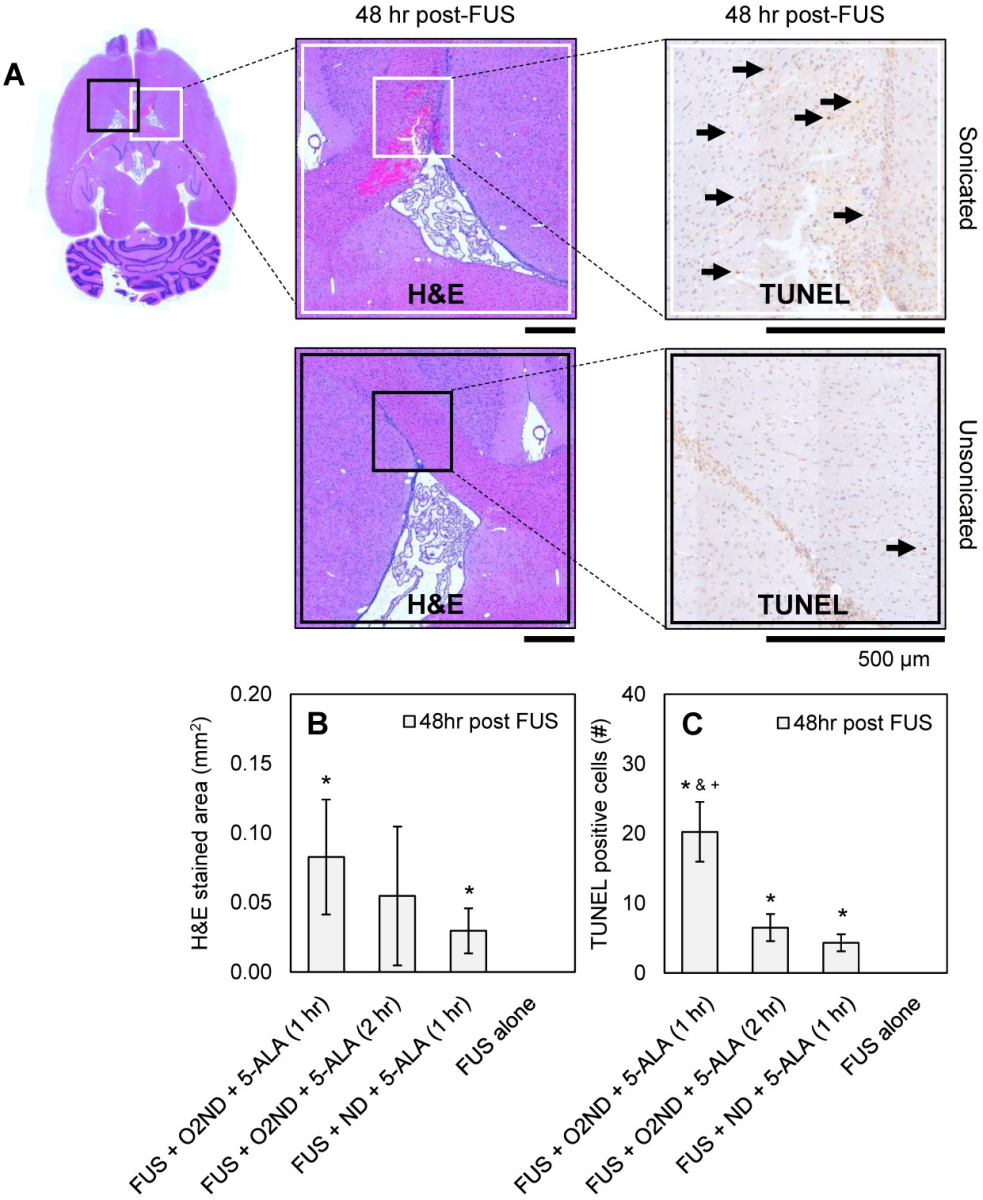
** Histological assessment of tissue damage following FUS treatment. (A)** Example histology from rat treated with FUS + O_2_ND + 5-ALA (1 hr). Scale bars show 500 µm. Damage from all rats is quantified by **(B)** haemorrhagic area on H&E-stained sections 48 hr post-treatment and **(C)** number of TUNEL positive cells 48 hr post-treatment. Each column represents data from 4 rats (1 rat was excluded from FUS + ND + 5-ALA (1 hr)), and error bars represent SEM. Significance compared to FUS alone is indicated by * (p<0.05), significance compared to FUS + ND + 5-ALA (1 hr) is indicated by & (p<0.05), and significance compared to FUS + O_2_ND + 5-ALA (2 hr) is indicated by + (p<0.05).

48 hr after sonication, rats were perfused, H&E-stained sections were used to identify haemorrhagic regions, and TUNEL-stained sections were used to identify apoptotic cells. Figure 4A shows representative histology images from a rat treated with FUS + O_2_ND + 5-ALA (1 hr), with magnified regions showing sonicated (white box) and unsonicated (black box) tissue. A greater number of apoptotic cells were seen around haemorrhagic regions. Haemorrhagic regions identified with H&E staining were greatest for rats exposed to FUS + O_2_ND + 5-ALA (1 hr) (Figure 4B). FUS alone did not result in any detectable regions of haemorrhage or increased apoptosis. The number of TUNEL positive cells in the sonicated striatum was significantly greater for FUS + O_2_ND + 5-ALA (1 hr), compared to FUS alone, FUS + ND + 5-ALA (1 hr), and FUS + O_2_ND + 5-ALA (2 hr) (Figure 4C).

Overall, both MRI and histological assessments showed large variability in severity and extent of tissue damage within each exposure group. This is likely a result of using a fixed transmit pressure, not accounting for differences in skull thickness or cavitation activity, discussed further in the following sections.

### Acoustic monitoring

**Figure 5:**
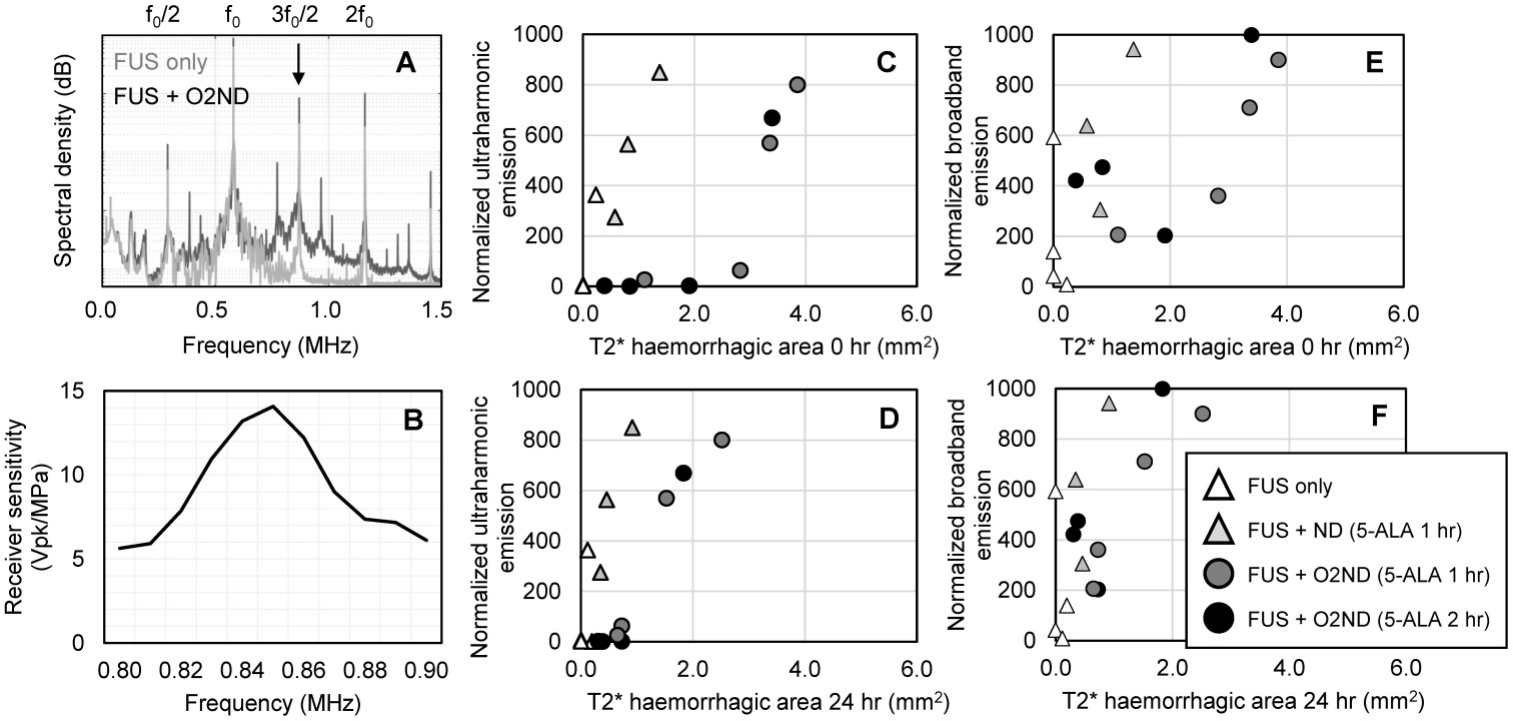
** Cavitation emissions from vaporizing nanodroplets. (A)** Representative spectral emissions during FUS alone and with oxygen-loaded nanodroplets show an increase in ultraharmonic and broadband emissions. **(B)** Sensitivity profile of the narrowband hydrophone. **(C)** Ultraharmonic (3f_0_/2) emissions mapped to haemorrhagic area immediately following sonication and **(D)** 24 hr post-sonication. **(E)** Broadband emissions mapped to haemorrhagic area immediately following sonication and **(F)** 24 hr post-sonication.

During sonication, cavitation emissions from vaporizing nanodroplets were monitored using a hydrophone tuned to the ultraharmonic frequency. As expected, the spectral emissions from unloaded nanodroplets and oxygen-loaded nanodroplets when exposed to FUS exceeded emissions detected from FUS alone (Figure 5A), detected with a narrowband receiver (sensitivity profile Figure 5B). Using a fixed transmit pressure of 2.0 MPa resulted in large variance in the magnitude of acoustic emissions and tissue damage. However, by isolating the magnitude of the ultraharmonic emission, cavitation activity could be mapped to the amount of tissue damage (Figure 5C, 5D). Broadband emissions were also greater in the presence of vaporizing nanodroplets compared to FUS alone (Figure 5E, 5F). T2* haemorrhagic area had a stronger correlation with ultraharmonic emissions (R^2^ = 0.95 +/- 0.06) than broadband emissions (R^2^ = 0.55 +/- 0.30) (assessed with exponential regression and averaged across the 4 experimental groups) and therefore may be a better predictor of SDT-induced damage. For the equivalent ultraharmonic emissions, oxygen-loaded nanodroplets achieved a greater haemorrhagic area compared to unloaded nanodroplets (Figure 5C). This trend was found to persist after 24 hr post-sonication (Figure 5D), illustrating the potency of oxygen-loaded nanodroplets during FUS exposure with 5-ALA.

## Discussion

Here we show how oxygen-loaded PFC nanodroplets, in combination with the sonosensitizer drug 5-ALA, can be a tool to enhance ablation of healthy cerebral tissue in a rat. Nanoscale volatile droplets were formulated from precursor Definity microbubbles and enabled the injectate to be supersaturated with dissolved oxygen, achieving a 58% increase in dissolved oxygen content compared to oxygen-saturated water. Vaporizing oxygen-loaded nanodroplets in the presence of 5-ALA resulted in a significant increase in haemorrhagic area, edema area and cell apoptosis compared to ultrasound alone.

As described by Costley *et al.* 6, the theoretical basis for sonodynamic therapy depends on the production of ROS, requiring ultrasound and molecular oxygen in the presence of a sonosensitizer drug. However, the mechanisms driving the improved SDT effect seen in this study are not fully understood; namely whether it is driven by local changes in molecular oxygen concentration, or through cavitation, or a combination. Based on the 2 mechanisms for ROS production - (1) directly from ultrasound and (2) by sonoluminescence - it is conceivable that the current platform exploits both pathways and is enhanced further by the sonosensitizer. Firstly, oxygen release may support mechanism 1, since droplets can provide additional oxygen to the surrounding tissue 40. Secondly, ROS production is known to occur in the core of a cavitating microbubble 41 and supports mechanism 2, namely if additional oxygen is present in the bubble core during collapse, more ROS may be produced. Furthermore, droplets have been reported to scavenge oxygen during vaporization 16 which may be reduced by pre-loading the droplet. Therefore, an oxygen-loaded cavitation agent may utilize several routes to enhance SDT, and future studies will investigate the importance of each for nanodroplet-mediated ablation.

Solubility of oxygen in PFC is typically over 20 times that of oxygen in water 42. This is increased further at low temperatures and with a high ratio of electronegative CF_3_ groups to CF_2_ groups as found in DFB (C_4_F_10_), where electronegative CF_3_ groups are associated with gas solubility 42. In this study, PFC volume at a concentration of 2.1 +/- 0.2 x10^11^ p/mL of 250 +/- 8 nm diameter droplets gives 0.2% PFC volume. Our results show a comparable oxygen uptake with Johnson *et al.* (40), using a 2% dodecafluoropentane (DDFP, C_5_F_12_) emulsion, with 215 nm diameter. Since DFB (C_4_F_10_) has a higher ratio of CF_3_ to CF_2_ groups (1:1), compared to DDFP (2:3), oxygen solubility is higher for the equivalent PFC volume.

It is likely that oxygen begins diffusing out of the droplets upon injection into the bloodstream due to the concentration gradient. However, since the droplet shell reduces diffusion of dissolved gases 43, and the presence of DFB slows the diffusion of oxygen 44, we anticipate that additional oxygen at the ultrasound focus plays a role in enhancing SDT. Future work will look at how oxygen diffusion may be slowed further by optimizing lipid chain length 45, and how using an implanted oxygen probe 46 or measuring oxygen relaxation rate through T1-weighted MR imaging 47 could be used to measure *in vivo* oxygen content at the target site.

Recently, Zeng *et al.* 48 developed a polymer-shell perfluorohexane (C_6_F_14_)-core nanoparticle for ultrasound-triggered oxygen delivery, measuring the production of singlet oxygen after ultrasound exposure, and successfully impaired tumour growth in a murine 4T1 mammary carcinoma model. The presence of locally delivered oxygen was essential in improving treatment. B-mode imaging was used to detect nanoparticle response to ultrasound, but was not mapped to a therapeutic effect.

In the current study, using oxygen-loaded DFB nanodroplets, vaporized using transcranial focused ultrasound, more tissue damage was achieved for the equivalent magnitude of acoustic emissions, when compared to unloaded nanodroplets (Figure 5). The magnitude of ultraharmonic emissions, a spectral characteristic unique to bubble activity, corresponded to the size of haemorrhagic area, suggesting that acoustic emissions from nanodroplet vaporization could offer real-time treatment monitoring of SDT. Previously, broadband emissions have been detected with a narrowband receiver, and have been used to predict tissue damage volume 49. However, in the current study, ultraharmonic emissions were found to be a better predictor of SDT-induced damage. This finding may be related to the fact that inertial cavitation is not necessary for ROS production to enhance SDT (Shibaguchi *et al.*, 2011). Activating 5-ALA with ultrasound may produce sufficient levels of ROS (regardless of broadband activity), enhanced by the presence of oxygen-loaded nanodroplets, generating more damage for the equivalent magnitude of cavitation emission.

## Limitations and future directions

The current study has several limitations which should be addressed in future work. Firstly, the drug dose was 10 times greater than that used clinically to treat glioma (20 mg/kg). A 5-ALA dose of 200 mg/kg was used, based on previous studies in normal rat tissue 13. Loading the sonosensitizer onto the cavitation agent could help to reduce systemic toxicities and enhance local delivery, as has been used in nanoparticle 48 and microbubble 51 formulations. However, if the drug cargo is limited to the shell of the construct, as is typical in microbubble and droplet designs, the encapsulated drug quantity may be small. Furthermore, this loading strategy also dictates the physiochemical properties of the drug that can be loaded, such as small-molecule lipophilic agents 33, or requires the drug to be modified 51. This limitation may be overcome by using a double emulsion technique 52.

The authors acknowledge that translating the current nanodroplet dose (1.52 mL/kg) to patients would require further safety assessment and would need to be delivered by infusion. The lipid volume delivered in this nanodroplet formulation is 10 times more than the typical clinical dose of Definity (0.01 mg of lipid/kg). However, compared to other perfluorocarbon nanoemulsions that have reached clinical trials, such as the blood-substitute Oxygent, the concentration is much lower. The nanodroplet dose used in the current study contains 1000 times less PFC than Oxygent (1.8 g of PFC/kg), with a comparable size emulsion (200 nm diameter) and phospholipid shell. We have reported previously that DFB nanodroplets remain acoustically responsive in the rodent brain for a half-life of 8.4 ± 1.7 min 33. By labelling the nanodroplets with a lipophilic fluorophore, the biodistribution has been mapped, showing uptake predominantly in the liver after 24 hr, with no signs of toxicity or adverse tissue effects in histology 27,53.

Secondly, using a fixed ultrasound pressure resulted in a large range of cavitation activity across animals (Figure 5). This is likely a result of differing skull thickness between animals, meaning the *in situ* pressure at the target may be different for each animal 54, and the concentration of nanodroplets at the focus. Using an acoustic controller that modifies the sonication pressure in real-time based on the detected acoustic emissions - as used to control safe blood-brain barrier opening - an open-loop 39 or closed-loop 55 controller would reduce treatment variability and could be designed to optimize treatment efficacy. To quantify the acoustic response of vaporizing nanodroplets we used a narrowband hydrophone, highly tuned at 850 kHz (-6 dB bandwidth of 60 kHz). It is possible that spectral energy leaking from the harmonic and ultraharmonic contributed to the calculated broadband energy. Incorporating a broadband receiver in future studies will allow us to assess a wider range of acoustic emissions.

Thirdly, the sacrifice and perfusion time point was set at 48 hr post-treatment to capture regions of haemorrhage and apoptotic cells. However, previous studies have reported cell death to be significant at 3 days post-treatment. Nyamekye *et al.* 13 show depletion of normal endothelial cells at 3 days following sonication and 5-ALA injection, and regeneration of endothelial lining at 14 days. A time course study could be conducted to explore the progression of cell death in more detail. Future work will also explore using automated image processing to assess tissue damage and avoid any potential errors in manual contouring techniques 56.

Finally, 5-ALA is known to have very low blood-brain barrier permeability 57. Therefore, most SDT-mediated damage in normal cerebral tissue is localized to the endothelial lining and in close proximity to vessels. Opening the blood-brain barrier prior to 5-ALA delivery with focused ultrasound and microbubbles 58 could increase drug uptake, reduce the required systemic drug dose, and generate more homogeneous tissue damage throughout the ultrasound focus. Furthermore, in the context of cancer therapy, enhancing SDT with oxygen-loaded nanodroplets could increase the efficacy of treatment due to the sonosensitizer preferentially accumulating in tumour cells. Nanoscale perfluorocarbon emulsions have been shown to extravasate from leaky tumour vasculature 59,60, further enhancing the potency of this treatment platform by bringing ROS production in close proximity to 5-ALA-treated cells.

## Conclusions

Unlike PFC blood substitutes which remain intact in circulation, nanodroplets can vaporize in the presence of ultrasound, generating microbubbles and collapsing at high pressures. We report that loading volatile PFC nanodroplets with oxygen increases the severity of local haemorrhage, edema and cell apoptosis in combination with the sonosensitizer 5-ALA. We show that bubble formation and collapse from oxygen-loaded nanodroplets generate sufficient ROS to damage normal cerebral tissue within the ultrasound focus.

Real-time treatment monitoring during sonodynamic therapy is currently lacking in clinical practice. Here we show that cavitation emissions from vaporizing nanodroplets may be mapped to tissue damage. Oxygen-loaded PFC nanodroplets were shown to be a useful tool to enhance and monitor sonodynamic therapy in the brain.

## Figures and Tables

**Table 1:** Distribution of animals between treatment groups, focused ultrasound (FUS) alone, or in combination with unloaded nanodroplets (ND), oxygen-loaded nanodroplets (O_2_ND), and the sonosensitizer 5-aminolevulinic acid (5-ALA).

Treatment group	N
FUS alone	4
FUS + ND + 5-ALA (1 hr)	4
FUS + O_2_ND + 5-ALA (1 hr)	4
FUS + O_2_ND + 5-ALA (2 hr)	4
